# A Resistance-Evading Antibiotic for Treating Anthrax

**DOI:** 10.21203/rs.3.rs-3991430/v1

**Published:** 2024-03-25

**Authors:** Dallas Hughes, William Lawrence, Jennifer Peel, de Winter Rosan, Losee Ling, Nitti Niiti, Peoples Aaron, Rhythm Shukla, Harold MacGillavry, Henry Heine, Hensel Martha, Whorton Elbert, Markus Weingarth, Kim Lewis

**Affiliations:** NovoBiotic Pharmaceuticals; University of Texas Medical Branch; University of Texas Medical Branch; Utrecht University; NovoBiotic Pharmaceuticals; NovoBiotic Pharmaceuticals; NovoBiotic Pharmaceuticals; AMOLF; Utrecht University; University of Florida; University of Texas MD Anderson; University of Texas Medical Branch; Utrecht University; Antimicrobial Discovery Center, Department of Biology, Northeastern University

## Abstract

The antimicrobial resistance crisis (AMR) is associated with millions of deaths and undermines the franchise of medicine. Of particular concern is the threat of bioweapons, exemplified by anthrax. Introduction of novel antibiotics helps mitigate AMR, but does not address the threat of bioweapons with engineered resistance. We reasoned that teixobactin, an antibiotic with no detectable resistance, is uniquely suited to address the challenge of weaponized anthrax. Teixobactinbinds to immutable targets, precursors of cell wall polymers. Here we show that teixobactinis highly efficacious in a rabbit model of inhalation anthrax. Inhaling spores of *Bacillus anthracis* causes overwhelming morbidity and mortality. Treating rabbits with teixobactinafter the onset of disease rapidly eliminates the pathogen from blood and tissues, normalizes body temperature, and prevents tissue damage. Teixobactinassembles into an irreversible supramolecular structure of the surface of *B. anthracis* membrane, likely contributing to its unusually high potency against anthrax. Antibiotics evading resistance provide a rational solution to both AMR and engineered bioweapons.

## Introduction

We are living at the time of the Antimicrobial Resistance (AMR) Crisis, a slow-moving pandemic^1^. The death toll associated with drug-resistant bacteria is 5 million world-wide^2^. The main cause of this pandemic is well understood – actinomycetes, the traditional source of antibiotics, have been overmined, leading to the collapse of the only reliable platform for discovery we had^3^. Not surprisingly, decades without the introduction of novel antibiotics and a persistent spread of resistant pathogens have led to the AMR Crisis. At the same time, there have been a number of encouraging advances over the past decade. Screening outside of actinomycetes resulted in the discovery of several novel antibiotics that are in development. These include natural products teixobactin^4^ and clovibactin^5^, cell wall synthesis inhibitors from previously uncultured *Eleftheria*; odilorhabdin^6^, a ribosomal inhibitor, and darobactin^7^ and dynobactin^8^, inhibitors of BamA from *Xenorhabdus* and *Photorhabdus*, symbionts of the gut microbiome of nematodes. Among the synthetic compounds, novel inhibitors of β-lactamases are being introduced, restoring activity to β-lactams^3^; a screen of macrocyclic peptides resulted in zosurabalpin, an inhibitor of LPS transport^9,10^; and the first rationally designed antibiotic, an inhibitor of penicillin-binding proteins, has been recently reported^11^. The rules governing penetration of compounds into bacteria are being deciphered^12,13^, which will help synthetic chemistry efforts. Teixobactin and clovibactin are especially relevant to the problem of AMR. These antibiotics bind to Lipid II and Lipid III, precursors of peptidoglycan and wall teichoic acid, and are free of detectable resistance development. Both compounds share another unusual property – once bound to the target, they form a supramolecular structure^5,14^, a striking departure from the conventional one compound/one target mechanism for all known antibiotics.

Of particular concern is the threat of engineered resistance, bioweapons. *Bacillus anthracis* is one of the most dangerous biodefense threats^15^, it is a Category A Priority Pathogen under the NIAID Emerging Infectious Diseases/Pathogens program. As the NIAID research agenda for CDC Category A Agents points out, historical rates for mortality of naturally occurring inhalation anthrax infections are very high, around 75%. The pathogen is stable in the form of spores, can be easily produced in large amounts, can be effectively delivered in the form of an aerosol, and is highly virulent. Release of *B. anthracis* from a small leak due to an improperly installed filter from a bioweapons facility in Sverdlovsk in 1979 gives us a preview of the devastation it can cause. 70–100 people died of inhalation anthrax, though the Soviet authorities at the time attributed the deaths to a natural cause, consumption of contaminated meat. A subsequent field study led by Matthew Meselson determined that the cause of the epidemic was release of spores from the facility down-wind^16^.

Ciprofloxacin, levofloxacin, penicillin and doxycycline are currently approved for antimicrobial prophylaxis and treatment of *B. anthracis* infection^17^. Additional antibiotics are being introduced to treat anthrax as well. For example, BARDA is stockpiling omadacycline^18^, an antibiotic from the tetracycline class, to treat anthrax in case of a bioterror attack. Both older and newly-introduced antibiotics are expected to be of “dual-use”, for both regular and bioterror defense purposes. However, resistance to all of these antibiotics can be easily engineered or produced through selection. Indeed, engineered drug resistant *B. anthracis* has been created^19^. Dual use assumes that all antibiotics are prone to resistance development, an unavoidable limitation of countermeasures. We do not currently have a reasonable response to engineered drug-resistant bioweapons. The discovery of teixobactin and clovibactin challenges this old paradigm and provides us with an opportunity to develop countermeasures to bioweapons that are not susceptible to resistance.

In this study, we undertake a detailed evaluation of teixobactin in treating inhalation anthrax in a rabbit model of the disease.

## RESULTS

### In vitro characterization of teixobactin against B. anthracis.

**Figure 1:**
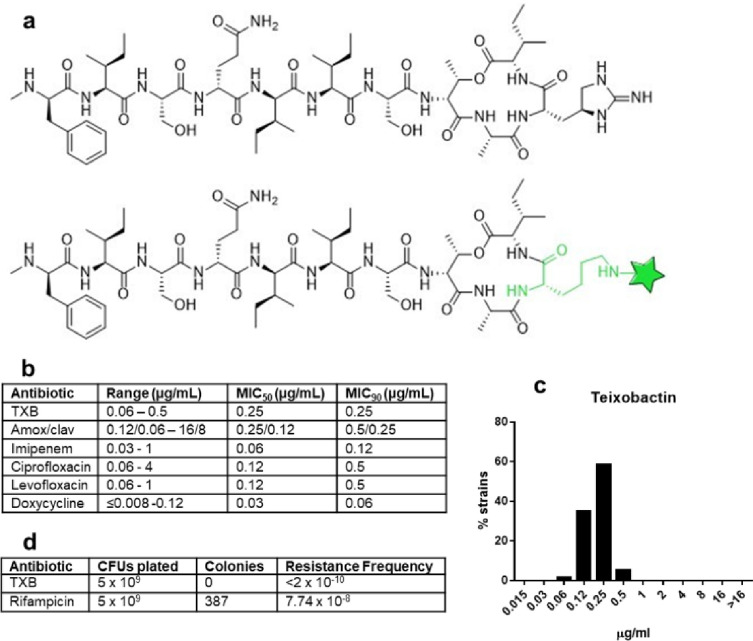
In vitro activity of teixobactin(TXB) against *B. anthracis*. **a**, Teixobactin and fluorescent Lys(Bodipy FL)10-teixobactin. **b**, MIC was determined in cation-adjusted Mueller Hinton broth (CA-MHB) plus 0.002% polysorbate 80 by broth microdilution following CLSA guidelines. **c**, TeixobactinMIC with 30 tested strains follows a Gaussian distribution. **d**, Resistance frequency determined by plating spores of the *B. anthracis* Ames on nutrient agar and counting colonies (CFU, colony forming units). Rifampicin is used as a control.

We previously reported that teixobactin (Fig. 1a) acts against *B. anthracis*^4 20^. The MIC was determined for a set of virulent strains from the NIH Biodefense and Emerging Infections Research Resources (BB Resources) repository that are commonly used in laboratory experiments, Ames (NR3838 and NR-41), Kruger B (NR-412), Vollum (NR-414), Graves (NR-41) and T CNEVA (NR-413). The MIC range for these strains was 0.06–0.125 μg/mL. In assessing developmental potential of a compound, it is important to determine MIC_90_, the concentration of antibiotic that inhibits growth of 90% of tested strains. For this, we took advantage of a collection of 30 pathogenic strains from different sources. Several antibiotics that have been used to treat anthrax are included for comparison (Fig. 1b; Supplemental Table 1). The teixobactin MIC_90_ is 0.25 μg/ml, suggesting a consistently high potency against pathogen isolates, comparable to the clinically available antibiotics. The distribution of MIC is Gaussian, and the difference between the main (0.25) and the highest MIC (0.5) is only twofold (Fig. 1c). By contrast, MIC_90_ of ciprofloxacin, the most commonly used antibiotic to treat anthrax, is 0.5 μg/ml, while isolates with a much higher MIC of 4 μg/ml are present, suggesting resistance. The minimal bactericidal concentration (MBC) for the Sterne strain of *B. anthracis* was 0.125 μg/mL, twice the MIC.

Teixobactin is characterized by the lack of detectable resistance, which was studied in detail with *Staphylococcus aureus*^4^. No resistance was observed with other pathogens as well, such as *Mycobacterium tuberculosis*. Consistent with this, plating *B. anthracis* on nutrient agar medium containing a fairly low level of teixobactin, 1 μg/ml (4X MIC), produced no colonies, with a calculated frequency of resistance < 2 × 10^−10^ (Fig. 1d). By contrast, plating rifampicin at a relatively higher concentration, 16X MIC, produced resistant colonies at a frequency of 7.74 × 10^− 8^. Rifampicin resistance is well-defined and conferred by mutations in the target, RNA polymerase, making it a convenient control antibiotic in resistance experiments.

Given the consistently high potency, excellent bactericidal activity, and lack of detectable resistance to teixobactin, we next examined its ability to treat an infection in an animal model of anthrax.

### In vivo efficacy

Rabbits serve as the standard model for assessing efficacy of therapies against anthrax, since the clinical manifestation of the disease most closely resembles that of humans^21^. In a preliminary study, we found that teixobactin at 5 mg/kg cleared the infection in rabbits, but also caused hyperthermia, likely due to the disruption of the gut microbiome which is common in rabbits ^20^. We therefore began with a pharmacokinetics (PK) analysis to determine minimal levels of the drug that can be efficacious. Weaponized anthrax is spread by spores and acquired through respiration. After the spores germinate in the lungs, the pathogen spreads throughout the body, and the infection is a typical bacteremia^22^. The presence of the drug in the blood then predicts treatment efficacy.

**Figure 2:**
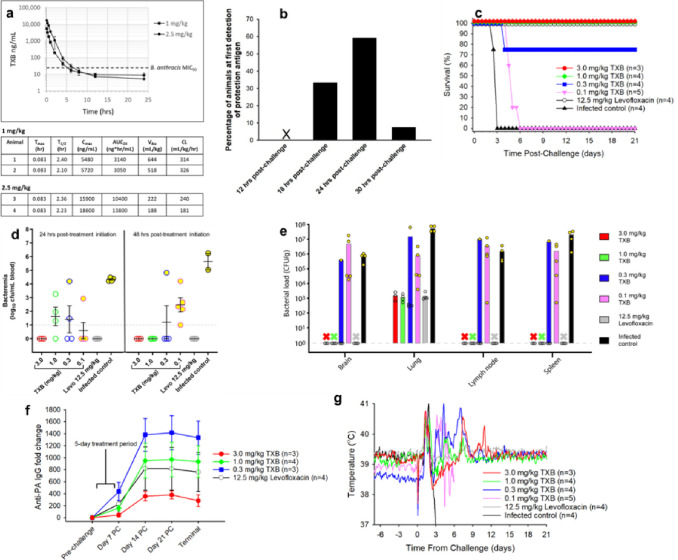
In vivo efficacy. **a**, Pharmacokinetic analysis of teixobactin (TXB) in rabbits after a single IV injection of either 1 mg/kg or 2.5 mg/kg. PK Parameters: T_max_ = time of maximum teixobactin concentration; T_1/2_ = time required for plasma concentration of teixobactin to decrease by 50%; C_max_ = the highest (peak) teixobactin concentration; AUC_24_ - area under the serum concentration vs. time curve representing the total teixobactin exposure over 24 hours; V_dss_ = steady-state volume of distribution representing teixobactin’s propensity to either remain in the plasma or redistribute to other tissue compartments; CL: the clearance rate of teixobactin. **b**, Appearance of protective antigen (PA) after infection with *B. anthracis* spores. Rabbits were challenged with 255 × LD_50_ of *B. anthracis* spores by inhalation. Beginning 12 hours post-challenge, blood samples were collected every 6 hours to test for the presence of PA based on binding to an anti-PA antibody. **c**, Efficacy of treating the infection with teixobactin. Antibiotic was administered SID for 5 days by IV once the level of PA became detectable, and survival monitored for 21 days from treatment initiation. **d**, Blood levels of pathogen. Blood samples were collected at various times after treatment initiation to assess the level of bacteremia. The blood samples were plated on blood agar plates and incubated at 37°C for 16–24 hours. Colonies were enumerated using an automatic colony counter. **e**, Bacterial load in tissues. Tissue samples were collected at the end of the post-infection monitoring period 21 days after treatment initiation for survivors and 3–6 days post-infection for the non-survivors. 3 out of 4 animals treated with 0.3 mg/kg teixobactin survived infection. No bacterial counts denoted as X (all survivors). Open circles represent survivors and yellow filled circles represent non-survivors (d,e) . **f**, Increase in the amount of serum anti-PA IgG. **g**, Core body temperature was recorded every 10 minutes using implanted data loggers. This data was used to calculate 2-hour moving averages for each animal, and the results were averaged among the animals in each group.

Teixobactin formulated in 5% dextrose was administered IV to male New Zealand white rabbits at either 1.0 mg/kg or 2.5 mg/kg. Plasma samples were taken at various time points, processed, and analyzed for the presence of teixobactin by liquid chromatography-mass spectrometry (LC-MS). PK analysis was performed with Watson LIMS 7.3, using a noncompartmental model^23^ (Fig. 2a). There was an approximately 3-fold increase in the maximal blood level of the compound (C_max_) and a 4-fold increase in area under the curve (AUC), a measure of total exposure, going from dosing level of 1.0 mg/kg to 2.5 mg/kg, indicating a more than proportional increase in blood residence time. At these doses, the antibiotic remained in the blood for several hours above the MIC.

In order to establish an infection, an aerosol of *B. anthracis* spores was prepared with a nebulizer, and rabbits were exposed to the pathogen using a head-only chamber. The infective dose was 2.55 × 10^7^ spores, 255 times the LD_50_ to ensure a rapid onset of infection^24^.

Respiration was monitored by plethysmography to measure the rate (volume/minute). The nebulizer was programmed to stop aerosol delivery when the volume carrying the target dose of pathogen was achieved. In order to quantify the actual dose delivered to each animal, the air from the chamber was sampled through a tube under vacuum feeding into a cylindrical biosampler containing culture medium. An aliquot from the biosampler was then plated for CFU counts. The actual mean dose was 2.55 × 10^7^ spores, very close to the 2.0 × 10^7^ target. After the pathogen spreads throughout the body, resulting in a systemic infection, it produces detectable amounts of protective antigen (PA), a toxin component, and this serves as a starting point for treatment^25^. To monitor PA, blood samples were collected from animals every 6 hours beginning 12 hours post-infection, and the whole blood was centrifuged to collect the serum. The sera were then analyzed using an anti-PA antibody (Fig. 2b). PA was detected as early as 18 hours post-infection and as late as 30 hours post-infection, and the PA serum concentrations ranged from approximately 100 to 7,100 pg/ml when initially detected. Antibiotic was then administered by IV once daily for 5 days, and survival was assessed for 21 days after initiation of treatment (Fig. 2c). There was complete protection from killing at 1 mg/kg of teixobactin (p < 0.05), an unusually low dose for an antibiotic, showing high potency of killing. There was also considerable protection from killing at 0.3 mg/kg (75% survival). Note by comparison the efficacious dose of levofloxacin, the standard of care, at 12.5 mg/kg. According to Yee and co-authors, a 12.5 mg/kg dose of levofloxacin administered to *B. anthracis*-challenged rabbits resulted in levofloxacin peak levels that were equivalent to that for a recommended 500-mg daily dose in humans^26^. We similarly sought to predict the likely human dose for teixobactin. For this, allometric scaling based on body surface area^27^ predicts a very low human equivalent dose of 0.32 mg/kg (1 mg/kg (efficacious dose in rabbits) X 0.32 (human dose equivalent conversion factor) = 0.32 mg/kg, or approximately 19 mg for a 60 kg human.

During the course of anthrax infection, bacteria germinate in lymphoid tissue and eventually enter the bloodstream resulting in bacteremia. From the blood, pathogens migrate into tissues. To directly assess the ability of teixobactin to neutralize *B. anthracis*, we measured the concentration of *B. anthracis* cells in the blood of treated animals by plate count using whole blood samples collected at various times post-infection. Over half the animals were bacteremic by 24 hours post-challenge; however, by 48 hours after treatment initiation, only animals within the two lowest dose treatment groups (0.1 mg/kg and 0.3 mg/kg teixobactin) remained bacteremic (Fig. 2d). Among these two low-dose groups, only one out of four animals in the 0.3 mg/kg teixobactin group was bacteremic at 48 hours post-treatment initiation, while all animals in the 0.1 mg/kg teixobactin group were bacteremic at the same timepoint. In fact, all surviving animals were cleared of pathogen by 48 hours, and they remained so for the remainder of the post-challenge period. All infected control animals were bacteremic by 24 hours post-treatment and at termination which occurred 2–4 days post-infection. In comparing the average bacterial levels in the blood, all antibiotic treatment groups exhibited lower levels of bacteremia relative to the control group at 24 hours post-treatment initiation (p < 0.05), and all but one antibiotic treatment group (0.1 mg/kg teixobactin) exhibited lower levels of bacteremia compared to the control group at 48 hours (p < 0.01).At the 48-hr post-treatment initiation timepoint, no colonies were produced from blood samples for the 1.0 mg/kg and 3.0 mg/kg teixobactin treated animals, showing eradication of the pathogen from the blood.

Effective treatment either stops bacterial migration from blood or kills bacteria after tissue infiltration. To determine the degree of bacterial clearance, tissue samples (brain, lung, mediastinal lymph node, and spleen) were collected from each animal at the end of the post-infection monitoring period 21 days after treatment initiation for surviving animals and 3–6 days post-infection for the non-surviving animals. The collected tissues were homogenized and plated onto blood agar plates for colony counts. Animals that succumbed to infection had *B. anthracis* present in all tissues, and the concentration ranged from 10^3^ to 10^7^ CFU/g (Fig. 2e). Notably, animals that were treated and survived the infection completely cleared the pathogen from their brain, lymph node and spleen tissues. Bacteria only remained in the lungs. This is expected, since ungerminated spores remain in the lungs many days after infection. By this time, animals (and people) mount a sufficient immune response that prevents relapse of anthrax^28^.

During active infection, the vegetative cells secrete “protective antigen” (PA) and “lethal factor” (LF), and as the pathogens expand, the amount of both rises. The PA-LF directly damages the host, and this is an important component of the infection to monitor. We therefore collected blood samples from rabbits at various times post-infection, and sera were assayed for anti-PA antibody levels via ECL. Teixobactin is administered after detection of PA, and by 7 days post-infection, the serum anti-PA IgG titers begin to increase (Fig. 2f). The level of anti-PA antibody was lower in the 3 mg/kg teixobactin group as compared to the 12.5 mg/kg levofloxacin group. Apparently, teixobactin quickly kills the pathogen prior to a massive release of toxin, diminishing the immune response. Since the toxin causes damage, teixobactin may be a better therapeutic than fluoroquinolone, a current standard of care.

While survival is the principal measure of the effectiveness for treating anthrax, the ability of therapy to control symptoms early on is important as well. Temperature increase is particularly prominent during *B. anthracis* infection, and it wanes only if bacteria are neutralized. We therefore continuously measured the core body temperature of the animals pre- and post-infection. For this, temperature was recorded with an implanted temperature data logger in the peritoneal. The normal body temperature for a rabbit is around 39°C. The body temperature was monitored every 10 minutes, and then average temperature for each 2 hour timepoint for all animals in a given group was plotted (Fig. 2g). All treatment groups exhibited an increase in temperature due to the bacterial infection approximately 1.5 days post-infection (time of infection, Day 0). The initial temperature elevation, up to 41°C, subsided in the antibiotic-treated groups by Day 3, but thereafter the temperatures of the antibiotic-treated animals had transient increases that persisted until Day 11. The average temperature of the infected control group declined rapidly by Day 2–3 which is typical during the agonal phase of anthrax infection. By Day 12, the average temperatures of the antibiotic-treated animals returned to baseline and remained so for the remainder of the post-challenge monitoring period. Altogether, these results demonstrate that teixobactin at low doses was effective at reducing fever by resolving the bacterial infection.

### Histopathology

**Figure 3:**
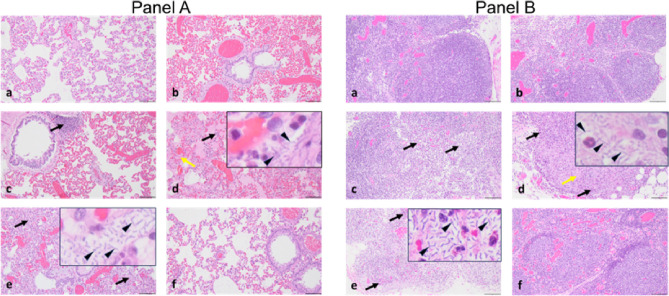
Histopathology of tissues. **Panel A, a-f**, Representative H&E-stained sections of lung tissue from rabbits challenged with a lethal dose of *B. anthracis* spores followed by treatment. **a**, 3.0 mg/kg teixobactintreatment. **b**, 1.0 mg/kg teixobactintreatment. **c**, .0.3 mg/kg teixobactintreatment (black arrow indicates congestion of the alveolar septa). **d**, 0.1 mg/kg teixobactintreatment (black arrow indicates inflammation in the alveolar septa, yellow arrow indicates fibrin deposition in the alveoli, and arrowheads indicate bacilli). **e**, Untreated (black arrows indicate inflammation in the alveolar septa and arrowheads indicate bacilli). **f**, 12.5 mg/kg levofloxacin treatment. Scale bars in a-f, 50 μm; scale bars for inserts in d and e, 10 μm. **Panel B, a-f**, Representative H&E-stained sections of mediastinal lymph nodes from rabbits challenged with a lethal dose of *B. anthracis* spores followed by treatment. **a**, 3.0 mg/kg teixobactin treatment. **b**, 1.0 mg/kg teixobactin treatment. **c**, .0.3 mg/kg teixobactin treatment (black arrows indicate heterophilic inflammation). **d**, 0.1 mg/kg teixobactin treatment (black arrows indicate heterophilic inflammation, yellow arrow indicates necrosis, and arrowheads indicate bacilli). **e**, Untreated (black arrows indicate inflammatory infiltrates and arrowheads indicate bacilli). **f**, 12.5 mg/kg levofloxacin treatment. Scale bars in a-f, 50 μm; scale bars for inserts in d and e, 10 μm.

To assess the degree of disease mitigation due to treatment with teixobactin, we collected tissues 21 days post-treatment initiation for the surviving animals and 3–6 days post-infection for the non-surviving animals. The tissues were fixed in formalin, processed for sectioning, and stained with hematoxylin and eosin. The lungs, lymph nodes, spleen, and liver showed the most notable damage in the unprotected animals (Fig. 3, Supplemental Fig. 1). The infected animals that were untreated showed characteristic changes of anthrax pathology, which included cellular and tissue degeneration, - necrosis, congestion, hemorrhage, edema, and inflammatory infiltrates (fibrinous, mixed cellular). The presence of bacteria was evident in multiple tissues, and especially in the mediastinal lymph node, spleen, lungs and liver. Animals receiving 0.1 mg/kg teixobactin had similar lesions and lesion severity as those observed in the infected control animals which suggest that this dose was not adequate to provide a therapeutic effect, in agreement with the level of pathogen burden. In contrast, all but one animal treated with 0.3 mg/kg teixobactin, and all of the animals treated with either 1.0 mg/kg or 3.0 mg/kg teixobactin showed mitigated anthrax-related lesions. The levofloxacin control group also shows mitigated lesions. These results show that teixobactin treatment at a dose concentration of as low as 0.3 mg/kg administered SID for 5 days reduced the pathology associated with anthrax infection.

Additional developmental studies. Testing experimental compounds for treating infections such as anthrax that have a high rate of mortality is unethical in humans. Accordingly, efficacy and safety are examined in animal models, which enables a safety assessment in Phase 1 human clinical trials. FDA approval of the drug is then based on the Animal Rule, which requires a regular Investigational New Drug (IND) submission based on safety and efficacy in animal models, and safety in a Phase 1 trial. To this end, we performed a number of IND-enabling studies with teixobactin. Apart from efficacy described in this paper, we performed extensive safety studies (Supplemental Table 2). These studies showed no significant inhibition of cytochromes P450, no mutagenicity or genotoxicity, no hemolysis, no production of toxic metabolites, and a good therapeutic window in rats and cynomolgus monkeys. Combined with excellent efficacy in the rabbit model of inhalation anthrax, these studies indicate that teixobactin is a promising clinical lead for development as an anti-anthrax measure.

### Delivery by inhalation

Intravenous delivery of teixobactin is suitable for patients with active disease that exhibit symptoms. For a prophylactic treatment, when there is risk of exposure to anthrax, a more convenient route of delivery would be beneficial. Teixobactin is a large molecule that is not orally available, but delivery by inhalation is a viable option. In order to evaluate this route of delivery, we performed a PK study in mice, where, unlike in rabbits, the passage from nares to lungs is unobstructed, and inhalation can be emulated by simply administering the drug in drops on the nares. PK data then shows whether sufficient amount of teixobactin passes from lungs into the blood to achieve efficacy against *B. anthracis*.

Female CD-1 mice were anesthetized, and teixobactin was instilled dropwise into the nares at 3 mg/kg and 12.5 mg/kg. Blood was collected at several time points, processed and analyzed for teixobactin. We also measured the levels of teixobactin in bronchoalveolar lavage fluid (BALF). Urea levels were measured in both plasma and BALF and used to calculate the teixobactin concentration in the epithelial lining fluid (ELF) of the lung.

With the high dose equivalent to levofloxacin (12.5 mg/kg), the concentration of teixobactin in the ELF remained very high, over 100 mg/ml for more than 3 days and was close to that for the lower dose of 3 mg/kg as well (Supplemental Fig. 2). Notably, plasma levels were above the *B. anthracis* MIC for at least 24 hours with the high dose (12.5 mg/kg), and for 4 to 5 hours with the low dose (3 mg/kg). We noticed that the plasma and ELF levels did not decrease as rapidly as when the drug is delivered IV, suggesting the ELF may be acting as a depot. The half-life (t_1/2_) for teixobactin delivered IV in mice is 1 to 2 hours, and 9.2 hours in this experiment. The PK results are encouraging and suggest that teixobactin will be efficacious in treating anthrax when delivered by inhalation. This will require formulating the compound for inhalation and testing in rabbits prior to evaluating in humans.

### Mechanism of action

**Figure 4:**
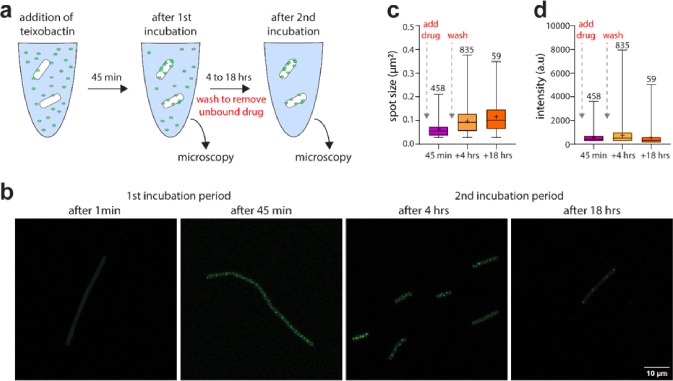
Formation of teixobactin supramolecular structures on the surface of the *B. anthracis* membrane. **a**, Illustration of the experimental setup to probe the formation and stability of teixobactin supra-structures with the *B. anthracis* Sterne strain. After the addition of a fluorescent teixobactin analogue^31^, bacteria were subjected to a first incubation period (1 or 45 min) and then imaged. Afterwards, cells were washed in buffer three times to remove unbound drug, then subjected to a second incubation period (4 or 18 hours), followed by imaging. **b**, Confocal microscopy of *B. anthracis* Sterne in the presence of a fluorescent teixobactin analogue. Images were acquired after the first (1 and 45 min) and second (4 and 18 hours) incubation periods. The images demonstrate that teixobactin forms suprastructures on the cell membrane and that these suprastructures, despite several washing steps, remain intact on relevant timescales of biological drug activity. Scale bar: 10 μm. **c,d**, boxplots of the **c**, fluorescent area and **d**, intensity of clusters after the different incubation steps. For each timepoint, combined data of a duplicate experiment are shown. The whiskers indicate the maximum and minimum value, the boxes indicate the 25^th^ and 75^th^ percentile, the (+) in the boxes show the mean and the lines show the medians. Numbers above each boxplots indicate the number of spots that contribute to the boxplot.

Teixobactin binds to Lipid II, precursor of peptidoglycan, and Lipid III, precursor of wall teichoic acid^29,30^. Once bound to its targets, teixobactin molecules assemble into a β-strand supramolecular structure on the surface of artificial phospholipid membranes and in *Bacillus megaterium*^5^. In order to probe formation of a supramolecular structure in *B. anthracis*, we used Lys(Bodipy FL)10-teixobactin^31^, (Fig. 1a), a fluorescent analog of teixobactin. After adding Lys(Bodipy FL)10-teixobactin to the Sterne strain of *B. anthracis*, formation of supramolecular structures is apparent by confocal microscopy imaging at the earliest time point examined of 45 min of incubation (Fig. 4a,b). Formation of supramolecular structures in which the relatively weak bonds linking teixobactin to its targets are additive may result in irreversible target engagement on relevant biological timescales. To test this intriguing possibility, we conceived an experimental approach in which, after initial formation of the teixobactin – Lipid II complex, all unbound teixobactin is removed, a scenario designed to favor drug – target dissociation and thus the unraveling of supra-structures. Here, after the initial incubation period of 45 min with Lys(Bodipy FL) 10-teixobactin, cells were thoroughly washed and resuspended in buffer three times to remove any unbound drug, followed by a second incubation period of 4 or 18 hours and subsequent imaging (Fig. 4a,b). Strikingly, washing had no apparent effect on the preformed supramolecular structures, showing that k_off_ (dissociation rate constant) of teixobactin is very slow in supra-structures that form irreversibly on relevant biological timescales (Fig. 4b–d). This irreversible action of teixobactin no doubt contributes to potent killing of *B. anthracis*.

## Discussion

Drug resistance diminishes the usefulness of even the best antibiotics. Based on the vast experience over the past 70 years of clinical use, resistance develops to all compounds^24^. The accepted response to this challenge is to introduce novel antibiotics to stay one step ahead of resistance. This strategy destines us to a never-ending battle with pathogens. This strategy does not work against engineered bioweapons. Novel compounds are developed for dual use, information on them is readily available, and it is fairly easy to select for, or engineer resistance *before* the compound becomes available for clinical use.

**Figure 5:**
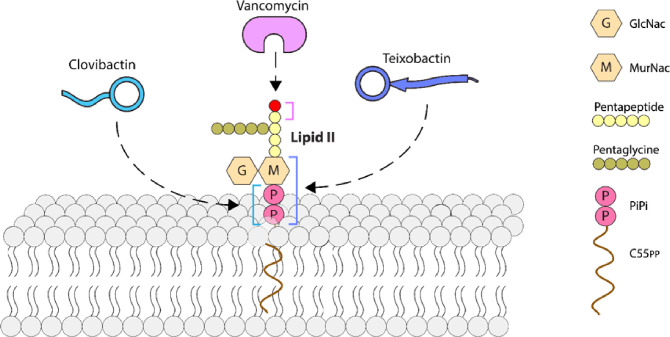
Progression to simplicity in binding moiety of resistance-evading antibiotics targeting Lipid II. Vancomycin binds to the pentapeptide that can be modified by a biochemical bypass replacing the terminal D-alanine with lactate (red); teixobactinbinds to sugar-PiPi, and clovibactinbinds to PiPi. Abbreviations: GlcNAcis N-Acetylglucosamine; MurNAcis N-Acetylmuramic acid.

Antibiotics differ quite a bit in their susceptibility to resistance development, and nature gives us important examples of evolution towards resistance evasion. In many if not most cases, diminishing resistance does not seem to have been under selection – for example, resistance to rifampicin is readily acquired by multiple mutations in the RNA polymerase. However, the best antibiotics in clinical use share an important property – multitargeting that diminishes the probability of resistance development and extends the useful life of a therapeutic^32^. Thus β-lactams target multiple homologous penicillin-binding proteins, and aminoglycosides bind to 16S rRNA that is encoded by multiple gene copies. As a result, resistance to these antibiotics usually stems not from target modification, but from acquisition of resistance genes that code for modifying enzymes, such as β-lactamases or aminoglycoside acetylases. A different type of resistance evasion is found in the recently discovered darobactin^7^. The antibiotic hits a single target, the BamA protein on the surface of the outer membrane, but binds to an essentially immutable moiety, the peptide backbone of a lateral gate with which incoming peptides interact prior to being folded by this chaperone into β-barrel proteins^33^. Indeed, substituting amino acids does not change the architecture of a peptide backbone. As a result, mutations conferring resistance to darobactin occur not in the binding site, but elsewhere in the protein, diminishing its fitness. The resistant mutants have a defective outer membrane and are avirulent, an interesting stealth mode of resistance evasion. Vancomycin provides a different, and more obvious example of binding to an immutable target. Vancomycin targets Lipid II, the precursor of peptidoglycan, binding to its pentapeptide moiety^34^ (Fig. 5).

Lipid II is not directly coded by genes, and from this perspective, the target is immutable. High-level resistance is conferred by a plasmid coding for a biochemical bypass, inserting lactate instead of D-alanine in the terminal position of the pentapeptide. The plasmid likely carries the resistance cassette from the producer or vancomycin, *Amycolatopsis orientalis*^35^. At the same time, this resilience is relative, low-level clinically significant vancomycin resistance does occur by mutations, for example, a thickened cell wall sequesters the antibiotic^36^. Teixobactin also binds to Lipid II, but to a simpler pyrophosphate-sugar moiety. The nature of the sugar is not essential, and teixobactin also binds to Lipid III, precursor of wall teichoic acid^4^. The producer is a Gram-negative *Eleftheria terrae*, and it protects itself from teixobactin by export across the outer membrane with a transenvelope pump that is part of the biosynthetic gene cluster. Teixobactin is a large compound and is restricted by the outer membrane of *E. terrae*. The target organisms are Gram-positive bacteria that cannot realistically borrow this mechanism of resistance from the Gram-negative producer. Another simple mechanism of resistance is target overexpression. However, teixobactin forms a supramolecular structure upon binding to Lipid II that damages the membrane, and adding more Lipid II will likely produce more damage. Clovibactin targets Lipid II and III as well but binds to what seems to be the simplest moiety possible – pyrophosphate^5^. There is little doubt that this is not modifiable, and clovibactin is likely the end of the road in the evolution of resistance evasion for Lipid II-type target binders. Both teixobactin and clovibactin have several layers of protection from resistance – multitargeting; binding to immutable targets, corrupting their targets, avoiding resistance by overexpression; and lacking a transferrable mechanism of resistance in the producer. Conceivably, resistance may evolve through a modifying enzyme, but these have only been described for antibiotics commonly present in the environment, such as β-lactams and aminoglycosides. No modifying enzyme emerged for the rare vancomycin since its introduction into the clinic some 70 years ago. Teixobactin and clovibactin are considerably rarer than vancomycin.

The rich diversity of resistance evasion strategies from nature points the way out of the AMR crisis, and provides a rational solution to the seemingly unsolvable problem of engineered bioweapons. In this study, we examined the potential of teixobactin for treating inhalation anthrax in a rabbit model of infection. Teixobactin is more advanced in development than clovibactin and is in late-stage IND-enabling studies at NovoBiotic. The studies included in vitro potency and cytotoxicity, mutagenesis and genotoxicity, metabolism, PK in rats and dogs, pivotal toxicity studies in rats, and 7-day toxicity in dogs. Teixobactin shows excellent potency against *B. anthracis*, with an MIC_90_ of 0.25 μg/ml, and MBC close to MIC. Excellent killing ability is especially important to treat anthrax, since the bacteria release toxins. An important feature of teixobactin is its ability to form a supramolecular structure upon binding to cell wall precursors, which damages the membrane, adding another target to its already complex mode of action^10^. We show that a supramolecular structure is formed on the surface of *B. anthracis* as well, and, importantly, its formation is irreversible on relevant biological timescales, which will contribute to the selectivity and high potency of this antibiotic. As with other bacterial species, we find no detectable resistance of *B. anthracis* to teixobactin.

Administration of teixobactin to rabbits infected by inhalation protects from the disease. Remarkably, the efficacious dose in the rabbit is 1 mg/ml, unusually low for antibiotics; the same therapeutic effect is achieved by the standard of care, levofloxacin, at 12.5 mg/kg. The predicted human dose is even lower, 0.32 mg/kg. Teixobactin effectively suppresses appearance of anti-PA antibodies, suggesting that rapid elimination of the pathogen limits the release of the damaging toxin. *B. anthracis* was effectively cleared both from the blood and tissues. Some pathogen remains in the lungs in the form of spores, but there was no relapse 21 days after treatment. Apparently, sufficient immune response builds up to protect from any germinating cells. Teixobactin also rapidly resolved symptoms, as determined by return of body temperature to normal. This is consistent with histology analysis, showing the lack of lasting damage for animals treated after the onset of infection.

The rabbit model of inhalation anthrax closely resembles human infection, and the results with teixobactin suggest that it is an excellent candidate for a novel therapeutic. A lengthy evolutionary process produced an antibiotic with a multilayer protection from resistance. We can now borrow this sophisticated weapon to counter a human-engineered menace.

## Materials and Methods

### Minimum inhibitory concentration (MIC)

was determined by the micro-dilution method in 96-well plates according to Clinical and Laboratory Standards Institute (CLSI formally NCCLS). Antibiotics were serially diluted twofold in 50 μl of CA-MHB. The antibiotic range was 16 to 0.008 μg/ml based on a final well volume of 100 μl after inoculation. For teixobactin wells, polysorbate 80 at a final concentration of 0.002 % was maintained. Plates were incubated at 35°C. MIC was determined visually at 18 hours.

Bacterial inoculum was prepared by suspension of colonies into cation-adjusted Mueller-Hinton broth (CA-MHB) from 18–24 hour *B. anthracis* Sheep Blood agar (SBA) plates that previously incubated at 35°C. Suspended cultures were each diluted with CA-MHB to a bacterial cell density of 10^6^ CFU/ml adjusted based on OD_600_. Conversion factor for *B. anthracis*; 3.82 × 10^7^ CFU/ml/OD. To each well of the 96-well plates, 50 μl of the adjusted dilution was added for a final inoculum of approximately 5 × 10^4^ CFU/well.

Quality control of antibiotic stocks was established by using *E. coli* ATCC 25922 and *S. aureus* ATCC 29213. Inoculums prepared as described above from 18–24 hour SBA plates. Conversion factors; *E. coli* 6.83 × 10^8^ CFU/ml/OD, *S. aureus* 2.07 × 10^10^ CFU/ml/OD.

### Minimum bactericidal concentration (MBC)

*B. anthracis* Sterne cells from the wells of an MIC microbroth plate incubated for 20 hours at 37°C were pelleted. An aliquot of the initial inoculum for the MIC was similarly processed. The cells were resuspended in fresh media and plated onto CA-MHA. The colonies were enumerated after incubating for 24 hours at 37°C. The MBC is defined as the first drug dilution that resulted in a 99.9% decrease from the initial bacterial titer of the starting inoculum. Experiments were performed with three biological replicates.

### Resistance Studies

For single step resistance, 5 × 10^9^ CFU of *Bacillus anthracis* Ames spores were embedded into CA-MHA containing either 1 mg/mL teixobactin (4 X MIC) or 8 mg/mL rifampin (16 X MIC). Colonies were enumerated after 4 days incubation at 37°C.

### Acquisition of Animals and Preparation

New Zealand White, specific-pathogen-free rabbits were obtained from Covance, Denver, PA. A venous access port (VAP) was surgically implanted into the external jugular vein of each animal by Covance to facilitate collection of multiple blood specimens. The VAP was also a convenient means of administering the appropriate test article I.V. during the study. Following VAP implantations and recovery, the animals were shipped to UTMB and housed in the Animal Resources Center (ARC) ABSL-2 animal housing facility. For this study, the animals were divided into two cohorts (equal number of rabbits per cohort) which arrived at UTMB approximately four weeks apart. Each cohort had an equal distribution of males and females. After arrival, the animals acclimated for at least 72 hours during which time each animal was given a thorough physical examination by a UTMB veterinarian. Following acclimation, each animal was implanted intraperitoneally with a DST micro-T temperature data logger (Star-Oddi Ltd) for the purpose of recording the animals’ temperature for the entire in-life period, after which the data were downloaded and analyzed. After recovery, the animals were transferred to the ABSL-3 facility within the Galveston National Laboratory (GNL) where the aerosol challenge and all subsequent experimental procedures were performed.

### Aerosol Challenge

While anesthetized (using ketamine/xylazine), the animals were challenged by aerosol with 200 LD_50_ of purified *B. anthracis* Ames NR3838 spores using an automated Biaera 3G aerosol control platform fitted to a muzzle-only aerosol chamber using a dedicated laptop computer to control, monitor, and record the humidity, pressures, and airflows. Real-time plethysmography was performed on each rabbit using a pair of DSI elastic band sensors placed around the animal’s thorax and abdomen, which were then calibrated with a pneumotach fitted to the face of each animal. After calibration, the rabbit’s muzzle was inserted into a Biaera aerosol chamber. The target dose of spores (D_P_) for aerosol deposition in the lungs was 2.0 × 10^7^ CFU. A 6-jet Collison nebulizer was used to generate the aerosol, which yields a spray factor (Sf) of 1.0–3.0 × 10^−6^for *B. anthracis* spores in our aerosol system. The nebulizer concentration to deliver this dose was calculated using standard algorithms, combined with a standard volume of air to deliver the target challenge dose of spores. Aerosol samples were collected continuously using an all-glass aerosol Bio-sampler (SKC, Inc.) for each exposure to confirm the challenge dose of spores for each animal by serial dilution and plating on TSA II with 5% sheep’s blood agar plates. The duration of aerosol delivery was based on the respiration rate of each animal and the total volume of inspired air monitored by the Biaera aerosol system computer.

### Clinical Observations

During the in-life phase of the study, clinical observations were performed/recorded at least twice daily, with more observations being performed during the peak time of infection when the animals typically show increased signs of infection. The animals’ weights were recorded daily starting upon placement on the study and continued for 14 days post-challenge. Thereafter, the animals’ weights were recorded weekly until the end of the in-life period of the study. Any animal found to be immobile (severe lack of movement when prodded/stimulated), unable to get to food/water, and/or in respiratory distress (abdominal breathing, open mouth breathing, nasal flaring) was immediately and humanely euthanized, and the time and date of death was recorded. At the time of euthanasia, terminal blood and tissue samples were collected. If an animal was found dead, the time and date of death was determined as accurately as possible, and terminal blood samples able to be recovered were collected. Lastly, all survivors were humanely euthanized on the last day of the in-life period, and all terminal samples taken. Survival was monitored for 21 days post-initiation of treatment (approximately 22 days post-challenge).

### Therapeutic Dosing

Teixobactin was formulated as weight per volume in 5% dextrose. Upon receipt, teixobactin powder was stored inside a desiccator at −20°C until formulation and treatment administration. Teixobactin was prepared as instructed by NovoBiotic. Briefly, teixobactin powder was dissolved in 5% dextrose and vortexed vigorously for 20 minutes. Subsequently, the solution was filter sterilized using 0.22 μm syringe filters (Fisher Scientific, cat# SLVVR33RS).

Starting immediately after a positive serum PA-ECL assay result post-challenge (trigger-to-treat), animals in Groups 1–4 were given teixobactin I.V., via the implanted VAP, at 3.0, 1.0, 0.3, and 0.1 mg/kg, respectively, once a day for 5 days. Group 5 (positive control group) was given levofloxacin I.V., *via* the implanted VAP, at 12.5 mg/kg once a day for 5 days beginning after a positive PA serum titer. Lastly, Group 6 (infected control group) was dosed with vehicle (5% dextrose) administered I.V., *via* the implanted VAP, once a day for 5 days beginning after a positive PA serum titer. All teixobactin doses were administered within 4 hours of formulation, and all treatment administrations (2 ml/kg) were performed as slow infusion (1–2 minutes).

The animals were not treated on an individual basis for the duration of the entire treatment period. The first dose administrations were prepared on an individual animal basis; however, all subsequent daily doses were prepared for treatment groups and delivered during the morning of each dosing day. The second treatment dose occurred in the AM of the next calendar day regardless of the time of initial treatment. All subsequent treatments occurred in the AM. For example, if Rabbit X was PA positive and initial treatment occurred at 03:00 hours on 28July2021, the next treatment was in the AM (~ 08:00 – 10:00) of 29July2022. Rabbit X was treated in the AM for the rest of the study.

### Blood Collection and Processing

Blood specimens were collected from each animal, *via* the VAP, prior to challenge and at specified times post-treatment initiation and post-challenge. Blood specimens were collected from the central ear artery instead of the VAP starting from day 7 post-challenge because *B. anthracis* in the blood of bacteremic animals had the potential to colonize the VAP (or catheter), thereby possibly contaminating successive samples drawn from the port. The impact of catheter colonization could have resulted in reporting an animal as bacteremia positive even though the blood had been cleared of bacteria by antibiotic treatment. Blood specimens were collected into Wampole blood collection microtubes for quantitative bacterial plate counts. Blood was also collected in serum separator microtubes and the serum used for PA-ECL and anti-PA IgG titration. Sample collection for PA quantitation occurred pre-challenge and every 6 hours beginning 12 hours post-challenge and continued until PA was detected, while sample collection for IgG quantitation occurred at −7, 7, 14, and 21 days post-challenge (and terminal).

### Assessment of Bacteremia and Bacterial Load

Bacterial concentration in the blood was determined using an automatic serial diluter and plater (easySpiral Dilute; Interscience). Whole blood, diluted in sterile water, was plated onto trypticase soy agar II plates containing 5% sterile sheep blood (TSAB) and incubated at 37°C for 16–24 hours. Colonies from the plates were then enumerated using an automatic colony counter (Scan 500; Interscience). Bacterial colonies having morphology typical of *B. anthracis* were subcultured and confirmed as *B. anthracis* with bacteriophage . Bacterial/spore load was also determined in lung, lymph node (mediastinal), brain, and spleen. These tissues were homogenized in a known volume of sterile water using a Stomacher 80 MicroBiomaster tissue homogenizer (Seward Ltd), and the homogenate was serially diluted in sterile water and plated onto TSA II with 5% sheep’s blood agar plates using the automatic diluter/plater (easySpiral Dilute, Interscience) and incubated at 37°C for 16–24 hours. Colonies from the plates were then enumerated using an automatic colony counter (Scan 500; Interscience), and the bacterial load was presented as CFU per gram of tissue. Bacterial colonies having morphology typical of *B. anthracis* were subcultured and confirmed as *B. anthracis* with bacteriophage g.

### Detection of PA in Serum

*B. anthracis* PA was measured in serum using a rapid PA-ECL screening assay produced by MesoScale Discovery (MSD; Gaithersburg, MD). The assay utilizes a detection antibody in combination with specialized 96-well microtiter plates that contain electrodes coated with an anti-PA capture antibody to detect and/or quantify PA. Following processing and assay execution, the amount of light emitted in sample wells is used to directly measure the amount of PA present in the serum based on a recombinant PA standard curve run in parallel. To quantitate the levels of PA in each serum sample, a standard curve (0–100 ng/ml) was analyzed in parallel on each assay day. Test samples were assayed in duplicate. The concentration of each test sample was extrapolated from the standard curve. Values that exceeded or fell below the upper and lower limits of the standard curve were defined as greater than the upper limit of quantitation (>ULOQ) or less than the lower limit of quantitation (<LLOQ), respectively.

### Detection of Anti-PA IgG in Serum

Anti-PA IgG was measured in rabbit sera *via* electrochemiluminescence similar to the PA-ECL screening assay. Biotinylated recombinant PA83 (List Biological Laboratories, Inc.) was bound to streptavidin-coated plates (MSD) and used as the capture antigen. Detection was accomplished using SULFO-TAG labeled anti-rabbit antibody and read buffer (MSD). Sera were diluted 10^−3^ prior to measuring in order to have all samples within the limits of detection. To determine the fold increase in signal, the respective pre-challenge timepoint for each treatment group was used as the reference.

### Necropsies and Histopathology

Necropsies were performed by UTMB veterinarians on animals that succumbed to infection during the study and those that were euthanized in accordance with humane or scientific endpoint criteria. In addition to gross pathology, microscopic pathology was assessed on hematoxylin and eosin (H&E) stained sections of lung, mediastinal lymph nodes, brain, spleen, liver, kidneys, and heart, and evaluated by light microscopy. A four level severity scale was used when appropriate utilizing the following terms: minimal (1 of 4), mild (2 of 4), moderate (3 of 4), and marked (4 of 4). Histopathological processing and analysis were performed by the Keeling Center for Comparative Medicine and Research at The University of Texas MD Anderson Cancer Center in Bastrop, Texas.

### Pharmacokinetic analysis

Male New Zealand white rabbits (n=2) were injected intravenously with a single dose of either 1.0 mg/kg or 2.5 mg/kg teixobactin formulated in 5% dextrose through an ear vein catheter. Plasma samples (0.5 mL) were taken at 0.083, 0.25, 0.5, 1, 2, 4, 6, 8, 12 and 24 h postdose. An aliquot of plasma sample or calibration sample was mixed with an equal volume of acetonitrile/0.1% formic acid containing propranolol hydrochloride as an internal standard, incubated on ice for 10 min, and centrifuged. The protein-free supernatant was analyzed for teixobactin by LC-MS/MS using an AB Sciex API 6500 mass spectrometer coupled to a Waters Acquity UPLC HSS T3, 30 × 2.1mm ID, 1.8 mM particle with Phenomenex Krudkatcher column prefilter. Samples were separated using an acetonitrile water gradient system, and peaks were analyzed using ESI ionization in MRM mode. The product m/z analyzed was 134.1D. The mean teixobactin plasma concentration and standard deviation within each time point were calculated and plotted. Data analysis was performed to determine the time of maximum plasma concentration, half-life, peak plasma concentration, area under the plasma concentration curve, volume of distribution, and clearance (T_max_, T_1/2_, C_max_, AUC_24_, V_dss_ and CL) with Watson LIMS 7.3, using a noncompartmental model.

### Determination of teixobactin in the lung and plasma after intranasal delivery

Female CD-1 mice were acclimated for 5 days prior to start of study. Mice were anesthetized through isoflurane inhalation and a 50 μl volume of teixobactin formulated in 5% dextrose was delivered by holding the mice in a vertical plane and instilling dropwise onto the nares. At different timepoints, three mice per timepoint were euthanized by CO_2_ inhalation. Blood was collected through cardiac puncture into K_2_EDTA collection tubes and processed for plasma. Bronchoalveolar lavage fluid (BALF) was collected through two consecutive 0.5 mL saline flushes. The BALF and plasma were analyzed for teixobactin as described above. Bronchoalveolar lavage enables sampling the epithelial lining fluid (ELF) of the lower respiratory tract, but also results in a significant dilution of that fluid. To quantify the apparent volume of ELF obtained from the BALF, urea is used as an endogenous marker of ELF dilution. Urea in the BALF and plasma was measured using Quantichrom^™^ Urea Assay Kit (DIUR-100) from BioAssay Systems (Hayward, CA). Since urea diffuses readily through the body, plasma and in situ ELF urea concentrations are identical; thus ELF volume can be calculated using simple dilution principles by comparing urea levels in the BALF and plasma. Using this dilution factor, the concentration of teixobactin in the ELF can be calculated from its concentration measured in the BALF.

### *Bacillus anthracis* imaging with Lys(Bodipy-FL)_10_-teixobactin

#### Incubation protocol:

*Bacillus anthracis* Sterne 34F2 (*LLNL A0517*) was grown on an LB-plate overnight at 33 °C. Secondary culture was grown until an OD_600_ of 0.3 was reached. Four aliquots of 500 μl of culture were pelleted by centrifugation at 3000*g* for 5 min. (*First incubation period*) Two of the pellets were resuspended with 200 μl from a 1μg/ml stock of Lys(Bodipy FL)10-teixobactin and incubated for 1 or 45 minutes. Afterward, cells were washed for three times by pelleting and resuspension in 200 μl buffer (100 mM Na_2_HPO_4_ and 18 mM KH_2_PO_4_, pH 7.4). The cells were then fixed by 10 min incubation in 200 μl 4% formaldehyde. Fixed bacteria were pelleted and washed once before adding resuspended cells to solidified 75–100 μl 2% agarose pads and closed with coverslip. (*Second incubation period*) The other 2 pellets were also subjected to the same first incubation period of 45 min with Lys(Bodipy FL)10-teixobactin. Afterwards, to thoroughly remove all unbound drug, cells were washed three times in buffer and then incubated for 4 or 18 hours. Cells were then washed again, fixed and imaged. Imaging was done using a LSM 700 confocal microscope.

#### Imaging:

Z-stack of 9 slices, separated by 0.5 μm each, were acquired from the center of focus with the Zeiss LSM 700 confocal microscope with a ×63/1.2 NA oil objective lens. A 488nm laser was used to excite Lys(Bodipy FL)10-teixobactin. After acquiring microscopy data, each of the Z-stacks was compressed to one image showing maximum intensity. The FIJI plugin ComDet 0.5.5 (https://github.com/UU-cellbiology/ComDet) was used to select and analyze spots of fluorescent teixobactin. Selection parameters were set to include larger particles, having an approximate particle size of 3 pixels and intensity threshold (in SD) of 30, ROI shape: squares. Only bacteria that were completely in frame and non-overlapping were analyzed. Comdet 0.5.5 was used to acquire the number, area, and integrated intensity of the spots. Boxplots were made using GraphPad.

## References

[1] Tacconelli E. Discovery, research, and development of new antibiotics: the WHO priority list of antibiotic-resistant bacteria and tuberculosis. Lancet Infect Dis 18, 318–327, doi:10.1016/s1473-3099(17)30753-3 (2018). 29276051

[2] Antimicrobial Resistance C. Global burden of bacterial antimicrobial resistance in 2019: a systematic analysis. Lancet 399, 629–655, doi:10.1016/S0140-6736(21)02724-0 (2022). 35065702 PMC8841637

[3] Lewis K. The Science of Antibiotic Discovery. Cell 181, 29–45, doi:10.1016/j.cell.2020.02.056 (2020). 32197064

[4] Ling L. L. A new antibiotic kills pathogens without detectable resistance. Nature 517, 455–459, doi:10.1038/nature14098 (2015). 25561178 PMC7414797

[5] Shukla R. An antibiotic from an uncultured bacterium binds to an immutable target. Cell 186, 4059–4073 e4027, doi:10.1016/j.cell.2023.07.038 (2023). 37611581

[6] Pantel L. Odilorhabdins, Antibacterial Agents that Cause Miscoding by Binding at a New Ribosomal Site. Mol Cell 70, 83–94 e87, doi:10.1016/j.molcel.2018.03.001 (2018). 29625040

[7] Imai Y. A new antibiotic selectively kills Gram-negative pathogens. Nature 576, 459–464, doi:10.1038/s41586-019-1791-1 (2019). 31747680 PMC7188312

[8] Miller R. D. Computational identification of a systemic antibiotic for Gram-negative bacteria. Nature microbiology 7, 1661–1672, doi:10.1038/s41564-022-01227-4 (2022). PMC10155127 36163500

[9] Pahil K. S. A new antibiotic traps lipopolysaccharide in its intermembrane transporter. Nature 625, 572–577, doi:10.1038/s41586-023-06799-7 (2024). 38172635 PMC10794137

[10] Zampaloni C. A novel antibiotic class targeting the lipopolysaccharide transporter. Nature 625, 566–571, doi:10.1038/s41586-023-06873-0 (2024). 38172634 PMC10794144

[11] Durand-Reville T. F. Rational design of a new antibiotic class for drug-resistant infections. Nature 597, 698–702, doi:10.1038/s41586-021-03899-0 (2021). 34526714

[12] Richter M. F. Predictive compound accumulation rules yield a broad-spectrum antibiotic. Nature 545, 299–304, doi:10.1038/nature22308 (2017). 28489819 PMC5737020

[13] Geddes E. J. Porin-independent accumulation in Pseudomonas enables antibiotic discovery. Nature 624, 145–153, doi:10.1038/s41586-023-06760-8 (2023). 37993720 PMC11254092

[14] Shukla R. Teixobactin kills bacteria by a two-pronged attack on the cell envelope. Nature 608, 390–396, doi:10.1038/s41586-022-05019-y (2022). 35922513 PMC9365693

[15] Inglesby T. V. Anthrax as a biological weapon: medical and public health management. Working Group on Civilian Biodefense. JAMA 281, 1735–1745, doi:10.1001/jama.281.18.1735 (1999). 10328075

[16] Meselson M. The Sverdlovsk anthrax outbreak of 1979. Science 266, 1202–1208, doi:10.1126/science.7973702 (1994). 7973702

[17] Head B. M., Rubinstein E. & Meyers A. F. Alternative pre-approved and novel therapies for the treatment of anthrax. BMC Infect Dis 16, 621, doi:10.1186/s12879-016-1951-y (2016). 27809794 PMC5094018

[18] Steenbergen J., Tanaka S. K., Miller L. L., Halasohoris S. A. & Hershfield J. R. In Vitro and In Vivo Activity of Omadacycline against Two Biothreat Pathogens, Bacillus anthracis and Yersinia pestis. Antimicrob Agents Chemother 61, doi:10.1128/AAC.02434-16 (2017). PMC5404541 28223382

[19] Davis J. A. & Schneider B. R. The gathering biological warfare storm. (Praeger, 2004).

[20] Lawrence W. S. Teixobactin Provides Protection against Inhalation Anthrax in the Rabbit Model. Pathogens 9, doi:10.3390/pathogens9090773 (2020). PMC7558628 32971758

[21] Lawrence W. S. The physiologic responses of Dutch belted rabbits infected with inhalational anthrax. Comp Med 59, 257–265 (2009). 19619416 PMC2733296

[22] Savransky V., Ionin B. & Reece J. Current Status and Trends in Prophylaxis and Management of Anthrax Disease. Pathogens 9, doi:10.3390/pathogens9050370 (2020). PMC7281134 32408493

[23] Gabrielsson J. & Weiner D. Non-compartmental analysis. Methods Mol Biol 929, 377–389, doi:10.1007/978-1-62703-050-2_16 (2012). 23007438

[24] Yee S. B., Dyer D. N., Twenhafel N. A. & Pitt M. L. Transient lipopolysaccharide-induced resistance to aerosolized Bacillus anthracis in New Zealand white rabbits. Comp Med 63, 252–261 (2013). 23759528 PMC3690431

[25] Kobiler D. Protective antigen as a correlative marker for anthrax in animal models. Infect Immun 74, 5871–5876, doi:10.1128/IAI.00792-06 (2006). 16988266 PMC1594923

[26] Yee S. B., Hatkin J. M., Dyer D. N., Orr S. A. & Pitt M. L. Aerosolized Bacillus anthracis infection in New Zealand white rabbits: natural history and intravenous levofloxacin treatment. Comp Med 60, 461–468 (2010). 21262133 PMC3002106

[27] FDA, U. (ed U.S. Department of Health and Human Services Food and Drug Administration Center for Drug Evaluation and Research) (Rockville, MD, 2005).

[28] Friedlander A. M. Postexposure prophylaxis against experimental inhalation anthrax. J Infect Dis 167, 1239–1243, doi:10.1093/infdis/167.5.1239 (1993). 8486963

[29] Ling L. L. Erratum: A new antibiotic kills pathogens without detectable resistance. Nature 520, 388, doi:10.1038/nature14303 (2015). 25731174

[30] Homma T. Dual Targeting of Cell Wall Precursors by Teixobactin Leads to Cell Lysis. Antimicrob Agents Chemother 60, 6510–6517, doi:10.1128/AAC.01050-16 (2016). 27550357 PMC5075054

[31] Morris M. A. Visualizing the mode of action and supramolecular assembly of teixobactin analogues in Bacillus subtilis. Chem Sci 13, 7747–7754, doi:10.1039/d2sc01388f (2022). 35865902 PMC9258396

[32] Silver L. L. Multi-targeting by monotherapeutic antibacterials. Nature Reviews Drug Discovery 6, 41–55 (2007). 17159922 10.1038/nrd2202

[33] Kaur H. The antibiotic darobactin mimics a beta-strand to inhibit outer membrane insertase. Nature 593, 125–129, doi:10.1038/s41586-021-03455-w (2021). 33854236

[34] Bugg T. D. Molecular basis for vancomycin resistance in Enterococcus faecium BM4147: biosynthesis of a depsipeptide peptidoglycan precursor by vancomycin resistance proteins VanH and VanA. Biochemistry 30, 10408–10415 (1991). 1931965 10.1021/bi00107a007

[35] Marshall C. G., Broadhead G., Leskiw B. K. & Wright G. D. D-Ala-D-Ala ligases from glycopeptide antibiotic-producing organisms are highly homologous to the enterococcal vancomycin-resistance ligases VanA and VanB. Proc Natl Acad Sci U S A 94, 6480–6483 (1997). 9177243 10.1073/pnas.94.12.6480 PMC21075

[36] Gardete S. & Tomasz A. Mechanisms of vancomycin resistance in Staphylococcus aureus. J Clin Invest 124, 2836–2840, doi:10.1172/JCI68834 (2014). 24983424 PMC4071404

